# Performance of a targeted next generation sequencing assay for mycobacterial identification and drug resistance from sputum and isolates

**DOI:** 10.1128/jcm.00106-26

**Published:** 2026-06-12

**Authors:** Sharon K. Kuss-Duerkop, Paul Godo, Emily K. DeCurtis, Van Pham, Lorraine Abushanab, Valerie Archuleta, Gregory T. Robertson, Yongbao Wang, Reeti Khare

**Affiliations:** 1 Advanced Diagnostic Laboratories, National Jewish Health 2930, Denver, Colorado, USA; 2 University of Colorado Anschutz Medical Campus 129263, Aurora, Colorado, USA; 3 Mycobacteria Research Laboratories, Department of Microbiology, Immunology and Pathology, Colorado State University 3447, Fort Collins, Colorado, USA; 4 Department of Medicine, National Jewish Health 2930, Denver, Colorado, USA; University of Western Australia, Perth, Australia

**Keywords:** tNGS, mycobacteria, MTBC, tuberculosis, NTM, nontuberculous mycobacteria, susceptibility testing, targeted next generation sequencing, identification, genotypic susceptibilities, Deeplex, whole genome sequencing, molecular diagnostics

## Abstract

Accurate identification and genotypic drug resistance prediction in mycobacteria are essential for early diagnosis and appropriate clinical management. Targeted next-generation sequencing (tNGS) detects multiple genetic targets and enables direct testing from primary specimens. We evaluated identification using the Deeplex Myc-TB assay for *Mycobacterium tuberculosis* complex (MTBC) and nontuberculous mycobacteria (NTM) as well as MTBC drug-resistance prediction from 78 isolates and 102 sputum samples. DNA libraries were sequenced (Illumina MiSeq), and results were analyzed using defined acceptability criteria. Performance was compared against reference phenotypic and genotypic methods. Testing for MTBC was invalid in <2.5% of cases, but NTM testing was invalid more frequently, especially in sputum (>50%). Of the evaluable isolates/sputum, specificity was 100% for negative/nontarget organisms. Among isolates, sensitivity for organism identification was 92.7% (38/41) for MTBC, 100% (5/5) for rapidly growing mycobacteria (RGM), and 87.5% (14/16) for slowly growing mycobacteria (SGM). For sputum, sensitivity was 97.6% (41/42) for MTBC, 70% (7/10) for SGM, and 66% (2/3) for RGM. Categorical agreement of MTBC resistotypes against reference susceptibility methods was 91.9% (489/532) for isolates and 90.8% (472/520) for sputum, increasing to 96.8% (489/505) and 97.3% (472/485), respectively, when variants of unknown significance were excluded. Limit-of-detection studies confirmed published thresholds of 100–1,000 genomes/PCR in both isolates and sputum. Deeplex Myc-TB provides highly specific identification and reliable genotypic resistance profiling from isolates and sputum, especially for MTBC. While identification of rare NTM species and mixed infections remain challenging, tNGS demonstrates substantial promise for rapid mycobacterial diagnostics.

**IMPORTANCE:**

Rapid and accurate diagnosis of mycobacterial infections is essential for initiating effective treatment, interrupting transmission, and improving patient outcomes. Traditional phenotypic methods for organism identification and antibiotic susceptibility testing are slow and may be unreliable for certain drugs. This study evaluates the Deeplex Myc-TB targeted next-generation sequencing assay to identify mycobacteria and resistance-associated mutations from the *Mycobacterium tuberculosis* complex (MTBC) for both isolates and clinical sputum. Our findings show strong correlation of MTBC identification and resistance markers compared with reference methods.

## INTRODUCTION

Complete and accurate species- and subspecies-level identification of *Mycobacteriaceae*, such as those within the *Mycobacterium tuberculosis* complex (MTBC) or *Mycobacterium avium* complex (MAC), requires molecular methods (1, 2). In addition to identifying and differentiating closely related organisms, molecular assays can also detect mutations associated with antibiotic resistance. This application of genotypic susceptibility testing is valuable for mycobacterial testing because results can be produced in days, as opposed to weeks or months, when using traditional phenotypic antibiotic susceptibility testing (AST) methods (3).

Molecular multiplexing allows for simultaneous (sub)speciation and detection of drug resistance markers. Currently used assays, such as real-time PCR or line probe assays, are usually limited to a small number of targets and real-time PCR assays may not provide mutation level information (4). Next-generation sequencing (NGS) can overcome low multiplexing issues by examining a greater breadth and depth of genomic material, and mutations can be readily identified (3). The Deeplex Myc-TB Combo Kit (GenoScreen, France) is a high-throughput, targeted NGS (tNGS) assay that uses *hsp65* as a primary target, and *rrl* and *rrs* as secondary targets, for the identification of mycobacteria directly from sputum samples or cultured isolates (5). It also tests 18 genes commonly associated with drug resistance and reports a “resistotype” for 13 anti-tuberculosis antibiotics. This supports early detection of resistance which can improve patient outcomes (6). Bioinformatic analyses are performed on sequence data using the associated web application, which requires no computational biology expertise (7).

Here, we evaluated the performance of Deeplex Myc-TB to determine its ability to accurately identify members of the MTBC, as well as a broad range of nontuberculous mycobacteria (NTM), directly from sputum and from cultured isolates. In addition, we assessed its performance for genotypic resistance prediction in MTBC by comparing results with reference standard phenotypic antibiotic-susceptibility testing performed on corresponding isolates.

## MATERIALS AND METHODS

### Isolates

Isolates were acquired from BEI Resources, Belgian Coordinated Collection of Microorganisms (BCCM) Culture collections, American Type Culture Collection (ATCC), previously tested proficiency samples (CDC and Wisconsin State Laboratory of Hygiene), routine clinical samples, or laboratory-derived isolates. BCCM isolates used were ITM 500748 (Germany), ITM 500823 (Germany), ITM 500859 (Belgium), ITM 500861 (Belgium), ITM 501204 (USA), and ITM 501205 (USA). Note that there were limited MTBC strains available that were resistant to second-line drugs, so the number of resistant isolates tested was lower for antibiotics like capreomycin, bedaquiline, and linezolid. Testing was blinded, and blank isolates contained PrepMan Ultra Sample Preparation Buffer (4318930, Thermofisher Scientific, MA, USA) only. Non-target isolates included *Burkholderia gladioli*, *Corynebacterium* sp., *Enterobacter cloacae complex*, *Enterococcus faecalis* (two different clinical strains), *Escherichia coli*, *Gordonia* sp., *Morganella morganii*, *Staphylococcus aureus,* and *Staphylococcus lugdunensis*.

Two laboratory-derived isolates resistant to bedaquiline (BDQ) or BDQ/clofazimine (CFZ) were selected from wild-type *M. tuberculosis*, (WT Mtb) Erdman strain (ATCC 35,801). Mtb BDQ-R Erdman (MS146) was recovered as an *in vitro* escape mutant with an observed resistance frequency of 4.23 × 10^−8^ on in-house made Middlebrook 7H11 agar plates supplemented 0.2% [vol:vol] glycerol, 10% [vol:vol] oleic acid-albumin-dextrose-catalase (OADC) supplement, 0.01 mg/mL cycloheximide, and 0.05 mg/mL carbenicillin (7H11-OADC) and further supplemented with 0.5 µg/mL BDQ (PharmaBlock Sciences). Mtb BDQ/CFZ-R Erdman (MS199) was recovered as a spontaneous escape mutant on 7H11-OADC further supplemented with 0.5 mg/L BDQ and 0.25 mg/L CFZ (Sigma). MS199 was isolated from Mtb Erdman infected C3HeB/FeJ mouse lung tissue as a microbiologic relapse event 12 weeks following 16 weeks of treatment (5 of 7 days per week) with bedaquiline fumarate and pyrazinamide (Acros Organics), administered by oral gavage in 0.2 mL at 25 mg/kg and 150 mg/kg, respectively (to be published elsewhere).

### Smear and culture

Fluorescent smears for acid fast bacilli (AFB) were performed with TB Auramine O (Remel, Thermofisher, KS), and routine AFB culture was performed on solid and liquid media using standard methods (8). In brief, an equal volume of a 1% N-acetyl L-cysteine-sodium hydroxide solution was added to sputum samples. Each suspension was vortexed and incubated at room temperature for 15 min, followed by the addition of ~35 mL of phosphate buffer. Samples were centrifuged for 20 min at 3,500 *× g*, and the supernatant was discarded. Pellets were resuspended in 0.2% of bovine albumin solution. All processing and manipulation of blinded mycobacterial isolates and sputum samples were performed in a certified biosafety level 3 (BSL-3) laboratory.

### Specimens

Waste, post-processed sputum samples from routine clinical testing were selected if they were culture-positive for a range of mycobacteria. Since the frequency of culture-positive MTBC samples and less common NTM species was low, spiked MTBC (41/43) and NTM (5/32) samples were also included by adding acquired strains to artificial sputum matrix (ASM, BZ274, Biochemazone, Toronto, Canada) at various concentrations, including 2×, 5×, 7×, or 10× the limit of detection (LOD) (Table S9). Non-target samples contained the following organisms spiked into ASM at 10× LOD: *E. faecalis*, *Gordonia otitidis*, *Nocardia* sp. not further identified, *N. brasiliensis*, *N. cyriacigeorgica*, *N. farcinica*, *N. nova*, *N. veterana*, *N. otitidiscaviarum*, *S. aureus*, *Tsukamurella pulmonis*, or *T. tyrosinosolvens*.

### Targeted next-generation sequencing

**Fig 1:**
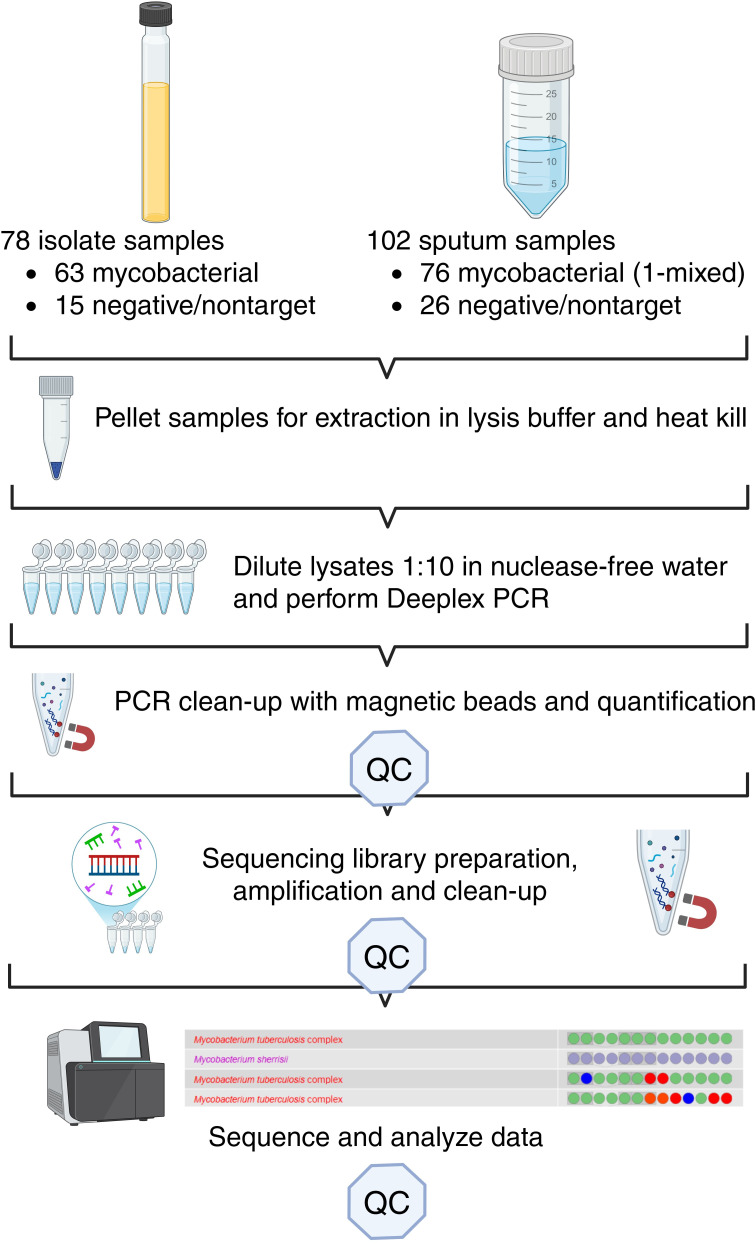
Workflow for isolate and sputum processing followed by Deeplex Myc-TB assay and analysis. Quality control (QC) checks were performed at the steps indicated to determine if samples passed or failed.

Crude DNA extraction was performed prior to tNGS as follows: Isolates were prepared by generating 0.5 McFarland suspensions. Isolate McFarland suspensions (1 mL) or processed sputum aliquots (1–2 mL) were centrifuged at 13,400 *× g* for 15 min, and the supernatant was discarded. Pellets were resuspended and vortexed in 200 µL of PrepMan Ultra Sample Preparation Buffer, heat inactivated at 95–100°C for 30 min, and then frozen at −20°C for at least 30 min (Fig. 1). Lysates were thawed and centrifuged at 17,000 *× g* for 15 min, and supernatants were transferred to a new tube. Each lysate was diluted 1:10 in nuclease-free water (Thermofisher) prior to use.

Testing was performed as outlined in the Deeplex Myc-TB protocol (7). In brief, Deeplex PCR was performed, and amplicons were cleaned (AMPure XP beads, Beckman Coulter, CA, USA or sparQ PureMag beads, Quantabio, MA). Amplicons were quantified on the Qubit 4 Fluorometer (Thermofisher) using the Qubit 1× or 2× dsDNA High Sensitivity quantification kits (Thermofisher). Amplicon libraries were generated with 1 ng of DNA (Nextera XT DNA Library Preparation Kit, Illumina, CA) and IDT for Illumina UD DNA/RNA indexes (Illumina). The sequencing libraries were quantified by Qubit, and nanomolar concentrations were determined. Libraries were pooled (2 nM total) in 10 mM Tris-HCl pH 8.5 (Bioworld, OH) containing 0.1% Tween20 (H5151, Promega, WI), denatured, and then diluted to 10 pM. PhiX control v3 (Illumina) was denatured and diluted to 12.5 pM, per the Denature and Dilute PhiX control for a 1% spike-in (Illumina). For the final sequencing reaction, 6 µL of the diluted/denatured PhiX was added to 594 µL of the 10 pM library pool and added to the MiSeq sequencing cartridge. Sequencing was performed on the MiSeq using the MiSeq Reagent Kit v2 300-cycles (up to 55 samples) or MiSeq Micro Reagent Kit v2 300-cycles (up to 25 samples) (Illumina). FASTQ files were downloaded from BaseSpace Sequencing Hub (BSSH, Illumina), uploaded to the Illumina Deeplex Myc-TB web application (euc1.platform.illumina.com/Deeplex; V3-0-1-Extended catalog) and analyzed using the 2nd ed. WHO catalog (Fig. 1).

### Range-finding and LOD

This was evaluated by generating 0.5 McFarland suspensions on a Sensititre nephelometer (Sensititre, Thermofisher, MA) of clinical Mtb and *M. abscessus* subsp. *bolletii* strains. Suspensions were diluted and tested in duplicate across five concentrations; the LOD was confirmed by testing ≥20 replicates of each organism and replicated by two independent operators for precision testing. Only sufficiently extracted isolates (Qubit value ≥0.2 ng/µL) were included. The mycobacterial suspensions were spot plated on 7H11 plates (Remel, Thermofisher, KS) and incubated at 37°C for up to 4 weeks to determine the actual quantification and amount per PCR.

### Reference methods for MTBC resistotyping

Phenotypic and genotypic testing was performed for each antibiotic as listed in Table S1. Bedaquiline susceptibility data from the BCCM Culture Collection were used as the comparator for Deeplex Myc-TB results for five isolates.

Agar proportion methods were performed on isolates for rifampin, isoniazid at low and high concentrations, streptomycin, ethambutol, capreomycin, amikacin, kanamycin, ethionamide, and bedaquiline as described (9). In brief, suspensions of MTBC that were ≤21 days old and were made in sterile demineralized water to a 0.5 McFarland using a nephelometer. Suspensions were diluted 100-fold into 0.02% tween solution, and 0.1 mL was inoculated into quadrants of 7H11 agar plates containing antibiotics at the relevant critical concentrations. Both susceptible (Vertulo strain) and resistant (Carnegie) MTBC control strains were used with every batch. See Table S1 for antibiotic concentrations that were tested. Growth was read at 14 days. The concentration at which <1% of growth compared to the growth control was recorded as being susceptible.

For clofazimine, agar dilution testing was performed where 7H11 agar quadrants contained serial dilutions of antibiotic, and each quadrant was inoculated with 0.1 mL of the diluted organism suspension as made above. The concentration at which no growth was detected was determined to be the minimum inhibitory concentration (MIC). As no clofazimine breakpoints exist for MTBC, ≤0.25 µg/mL was considered tentatively susceptible and an MIC of ≥0.5 µg/mL was considered tentatively resistant for the purposes of comparison to the tNGS results (10).

Broth proportion (11) was used on isolates for rifampin, isoniazid, pyrazinamide, ethambutol, streptomycin, capreomycin, kanamycin, ethionamide, fluoroquinolones (levofloxacin), and linezolid (Table S1). MTBC suspensions ≤21 days old were used to make 0.5 McFarland suspensions in sterile demineralized water. Suspensions were diluted (10-fold for antibiotic and 100-fold for control, per standard procedures) in sterile saline for BDQ and 0.02% tween solution for all other antibiotics; 0.5 mL of each suspension was used to inoculate Mycobacterial Growth Indicator Tubes (MGIT, BD Biosciences) with or without antibiotics. See Table S1 for antibiotic concentrations that were tested. Tests were considered interpretable if the growth control was positive within 4–13 days.

GeneXpert Mtb/Rif (Cepheid, Sunnyvale, CA) real-time PCR was performed on isolates to evaluate rifampin resistance in genotypic-phenotypic discrepants. Whole genome sequencing (WGS) was used for three Mtb isolates to examine mutations conferring BDQ resistance as follows: isolates were grown for 4–6 weeks at 37°C in 7H9 media (Thermofisher) to achieve dense culture growth (~10^8^ CFU/mL). Mtb DNA was extracted using the DNeasy PowerBiofilm Kit (24000-50, QIAgen, Germany) as previously described (12). In brief, ~1.9 mL of the mycobacterial suspension was centrifuged at 12,000 *× g* for 1 min, and supernatants were discarded. Bacterial pellets were resuspended in 500 µL of phosphate buffered saline (PBS, Thermofisher) and heat-killed at 95°C for 30 min. Suspensions were re-pelleted at 12,000 *× g* for 1 min, resuspended in 350 µL of Solution MBL, and transferred to PowerBiofilm Bead Tubes, and 100 µL of Solution FB was added. Samples were incubated at 65°C for 5 min, bead-beated for 1 min at 10,000 rpm using the Precellys Evolution (Bertin, France), and then processed according to the DNeasy PowerBiofilm protocol. Mtb DNA was eluted with 70 µL of EB, and sample concentrations and quality were checked on a Nanodrop ND1000 (Thermo Fisher). Cultures were also diluted and spot plated (10 µL) in triplicate on 7H11 plates for absolute quantification.

Library preparation was performed from 1 µg of Mtb DNA using the sparQ DNA Frag and Library Prep Kit (Quantabio, MA) by manufacturer’s protocol with the following modifications: (i) DNA was incubated for 8 min at 32°C to achieve ~300 base pair (bp) fragments. (ii) Optional library amplification was performed per manufacturer’s protocol for three cycles, purified using sparQ PureMag magnetic beads and quantified using the Qubit 1× dsDNA high sensitivity kit on the Qubit 4 (Thermofisher). DNA library fragment sizes were determined by Agilent TapeStation (National Jewish Health Sequencing Core, Denver, CO). Libraries were pooled (4 nM each), denatured, and diluted to 18 pM. For the final sequencing reaction, 6 µL of 12.5 pM PhiX was added to 594 µL of the 18 pM library and applied to the MiSeq reagent v2 300-cycle cartridge and sequenced on an Illumina MiSeq. FASTQ files were uploaded to BugSeq Bioinformatics, Inc. (BC, Canada) for analysis of short-read sequences using the BugRef Curated Database, which yields identification of species/subspecies and drug resistance variants.

Phenotypic testing was considered the true gold standard value unless discrepant results were evaluated using molecular methods (e.g., WGS for bedaquiline and real-time PCR for rifampin). If additional molecular methods were not available, isolates were evaluated using alternative phenotypic methods (e.g., broth proportion, broth microdilution, agar proportion, or antibiotic results provided by reliable vendors). In these cases, the consensus value of all the available results (i.e., majority) was considered the true value. If no alternative method was available (e.g., only single methods were available for pyrazinamide, fluoroquinolones, clofazimine, and kanamycin), testing was repeated using the same method, and phenotypic results were retained as the gold standard value. The details of primary and discrepant testing methods used for each antibiotic are specified in Table S1.

### Data analysis

Testing results were considered invalid if one or more of the following were true: (i) the Qubit score was <0.2 ng/µL after purification of Deeplex PCR amplicons (which results in <1 ng used for library preparation when 1 ng is needed per manufacturer’s instructions), (ii) the sequence quality score result was “ND” (mycobacteria not detected) for the identification of “ND” or “–” for resistotype, (iii) the sequencing Q30 score was <80%, or (iv) the average depth of coverage was <40× for identification and <600× for resistance variant detection. The total number of mycobacterial isolates or sputa that could be evaluated for accuracy purposes was determined by excluding the number of invalid samples from the total tested. Negatives or non-target organisms that were expected to yield invalid results were considered “evaluable” for the purposes of specificity analysis. Confidence interval calculations were performed using the GraphPad “Confidence interval of a proportion” (https://www.graphpad.com/quickcalcs/confinterval1/) web-based tool (MA, USA). Figure 1 was generated using BioRender.

## RESULTS

A total of 78 isolates were tested using Deeplex Myc-TB (42 MTBC, 5 RGM, 16 SGM, and 15 nontarget/negative) (Fig. 1). The overall invalid rate for mycobacterial isolates was 3.2% (2/63) (Table 1; Table S2); one *M. africanum* was invalid even upon repeat and was excluded from further accuracy analysis while one invalid *M. sherrisii* isolate was repeated with sufficient quality scores and could be included in downstream analysis. All (15/15) of the nontarget/negative samples were invalid (5 were blank and the remaining were spiked with non-mycobacteria). The most common cause was insufficient mycobacterial targets detected upon sequencing and analysis (Table S2). As this was the expected result, negative/nontarget isolates were considered evaluable for the purpose of specificity analysis.

**TABLE 1:** Invalid rate for the Deeplex-Myc-TB when testing isolates and sputum^*a*^

Organism	Isolates			Sputum		
	Total tested	# invalid due to failure of any criteria	Invalid rate	Total tested	# invalid due to failure of any criteria	Invalid rate
MTBC	42	1^*b*^	2.4%	43	1	2.3%
RGM	5	0	0%	11	8	72.7%
SGM	16	1^*c*^	6.3%	21	11	52.4%
Mixed	1	0	0%	1	0	0%
Negatives/nontarget organisms	15	15	100%	26	26	100%
TOTAL	79	17	N/A^*d*^	102	46	N/A

^*a*^ A test was invalid if one or more of the following were true: (i) Qubit score was <0.2 ng/µL after purification of Deeplex PCR amplicons, (ii) sequence quality score result was “ND” for identification or “ND” or “–” for resistotyping, (iii) Q30 score <80%, and/or (iv) average depth of coverage was <40× for identification and <600× for resistance detection (see Tables S1 and S2 for full details).

^*b*^ Testing was repeated but still had poor Quality (ND) and Qubit scores, likely due to poor growth of the isolate.

^*c*^ Repeat testing was performed and gave sufficient quality score upon repeat.

^*d*^ N/A, not applicable.

Out of 77 evaluable isolates, the overall sensitivity (as determined from correctly identified/evaluable isolates) was 91.9% (57/62) with a 95% confidence interval (CI) of 82.1%–96.9%, and overall specificity was 100% (15/15; 95% CI of 76.1%–100%). The percent agreement (as determined by the percent matching/evaluable isolates) per target type was as follows: 92.7% (38/41; 95% CI of 79.9%–98.2%) for MTBC, 100% (5/5; 95% CI = 51.1%–100%) for RGM, and 87.5% (14/16; 95% CI = 62.7%–97.8%) for SGM (Table 2).

**TABLE 2:** Accuracy of the tNGS assay for the identification of mycobacterial isolates*^i^*

Organism	Total tested	Total evaluable isolates^*h*^	Correctly identified	Incorrectly identified	Percent agreement	Confidence intervals
MTBC	42	41	38	3	92.7%	79.9–98.2
*Mycobacterium africanum*	2^*a*^	1	1	0	100%	
*Mycobacterium bovis*	3	3	3	0	100%	
*Mycobacterium bovis* BCG	4	4	2	2^*d*^	50%	
*Mycobacterium tuberculosis*	23	23	22	1^*e*^	95.7%	
*Mycobacterium tuberculosis* complex	10	10	10	0	100%	
RGM	5	5	5	0	100%	51.1–100
*M. abscessus* subsp. *abscessus*	1	1	1	0	100%	
*M. abscessus* subsp. *bolletii*	1	1	1	0	100%	
*M. abscessus* subsp. *massiliense*	1	1	1	0	100%	
*M. chelonae*	1	1	1	0	100%	
*M. fortuitum*	1	1	1	0	100%	
SGM	16	16	14	2	87.5%	62.7%–97.8%
*M. avium*	1	1	1	0	100%	
*M. chimera*	2	2	2	0	100%	
*M. gordonae*	1	1	1	0	100%	
*M. kansasii*	1	1	1	0	100%	
*M. conspicuum*	1	1	0	1^*f*^	0%	
*M. europaeum*	1	1	1	0	100%	
*M. florentinum*	1	1	1	0	100%	
*M. intermedium*	1	1	1	0	100%	
*M. intracellulare*	1	1	1	0	100%	
*M. malmoense*	1	1	1	0	100%	
*M. palustre*	1	1	1	0	100%	
*M. parascrofulaceum*	1	1	0	1^*g*^	0%	
*M. sherrisii*	1^*b*^	1	1	0	N/A	
*M. simiae*	1	1	1	0	100%	
*M. timonense*	1	1	1	0	100%	
Other	15	15^*c*^	15	0	100%	76.1%–100%
Negatives (Prepman only)	5	5^*c*^	5	0	100%	
Nontarget organisms	10	10^*c*^	10	0	100%	
TOTAL	78	77	72	5	93.5%	85.3%–97.5%

^*a*^ One excluded due to poor Qubit value, even after re-PCR and re-Qubit.

^*b*^ Originally failed due to poor Sequencing Quality Score (“ND”) but was acceptable upon re-PCR and re-Qubit.

^*c*^ As expected, all had inadequate depth of coverage and “ND” quality scores.

^*d*^ Deeplex resulted both as *M. bovis, *but repeat testing resulted in* M. bovis *BCG.

^*e*^ Deeplex resulted as “*M. tuberculosis* or *M. avium* or *M. canettii*” but additional colony types and *M. avium* was not detected in culture.

^*f*^ Deeplex resulted as *M. kansasii*.

^*g*^ Deeplex resulted as *M. seoulense.*

^*h*^ Evaluable isolates included total samples tested minus invalid samples.

^*i*^ N/A, Not applicable.

A total of 102 clinical or spiked sputum samples were also tested (Fig. 1). The invalid rate for the Deeplex Myc-TB assay tested directly on sputum was 2.3% (2/43) for MTBC, 72.7% (8/11) for RGM, 52.3% (11/21) for SGM, and 0% for the mixed sample (0/1), which contained *M. intracellulare* subsp. chimera, *M. abscessus* subsp. *abscessus,* and *Gordonia sputi* (Table 1). No samples were repeated due to sample depletion. Negative/nontarget samples consisted of 14 sputa with no organism and 12 containing aerobic actinomycetes and other non-mycobacteria. As expected, 100% (26/26; 95% CI = 84.8%–100%) of these negative/nontarget samples were invalid by one or more acceptability criteria (Table 1; Table S3). All negative/non-target samples were negative by AFB smear, including the sputa containing partially acid-fast organisms (Table 3).

**TABLE 3:** Correlation of culture, smear and tNGS sputum results

AFB culture		Smear		Evaluable by deeplex	
Result	Number	Result	Number (Percent)	Y (Percent)	*N*
Negative	16	Negative	16 (16%)	0 (0%)	16
Positive for partial acid fast	10	Negative	10 (10%)	0 (0%)	10
Positive for Mycobacteria	76	Negative	28 (27%)	16 (57%)	12
		1+	20 (20%)	14 (70%)	6
		2+	9 (9%)	7 (78%)	2
		3+	10 (10%)	10 (100%)	0
		4+	9 (9%)	9 (100%)	0
Total	102	N/A^*a*^	102 (100%)	56 (55%)	46

^*a*^ N/A, not applicable.

Of the sputum samples positive for mycobacteria by culture, 73.7% (56/76; 95% CI = 62.8%–82.3%) met all acceptability criteria and were considered evaluable by the Deeplex Myc-TB assay. Of all the culture-positive sputum samples, 63.2% (48/76; 95% CI = 51.9%–73.1%) were positive by smear, and the higher number of AFB detected in smears correlated with higher rates of evaluability (Table 3). The percent agreement per target was 97.6% (41/42; 95% CI = 86.6%–100%) for MTBC, 70% (7/10; 95% CI = 39.2%–89.7%) for SGM, 66.7% (2/3; 95% CI = 20.2%–94.4%) for RGM, and 0% (0/1; 95% CI = 0%–83.3%) for the mixed sample. An aggregate sensitivity across all sputum samples was not calculated, as the disproportionate representation of MTBC would artificially inflate the performance characteristics of the assay. Overall specificity was 100% (26/26; 95% CI = 84.8%–100%) (Table 4).

**TABLE 4:** Accuracy of the tNGS assay on for direct sputum^*c*^

Samples containing the following organisms	Total tested	Total evaluable isolates^*b*^	Correctly identified	Incorrectly identified	Percent agreement	Confidence interval
MTBC	43	42	41	1	97.6%	86.6%–100%
*Mycobacterium africanum*	2	1	1	0	100%	
*Mycobacterium bovis*	2	2	1	1	50%	
*Mycobacterium bovis* BCG	4	4	4	0	100%	
*Mycobacterium tuberculosis*	20	20	20	0	100%	
*Mycobacterium tuberculosis* complex	15	15	15	0	100%	
RGM	11	3	2	1	66.7%	20.2%–94.4%
*M. abscessus* subsp. *abscessus*	7	2	1	1	50%	
*M. abscessus* subsp. *massiliense*	3	1	1	0	100%	
*M. senegalense*	1	0	0	0	N/A	
SGM	21	10	7	3	70.0%	39.2%–89.7%
*M. avium*	3	1	1	0	100%	
*M. chimera*	2	0	0	0	N/A	
*M. gordonae*	1	0	0	0	N/A	
*M. intermedium*	1	1	1	0	100%	
*M. intracellulare*	11	6	4	2	66.7%	
*M. marinum*	1	0	0	0	N/A	
*M. parascrofulaceum*	1	1	0	1	0%	
*M. simiae*	1	1	1	0	100%	
Mixed	1	1	0	1	0%	0%–83.3%
*Mycobacterium chimera, Gordonia sputi,* and *Mycobacterium abscessus* subsp. *abscessus*	1	1	0	1	0%	
Other	26	26	26	0	100%	84.8%–100%
Negatives	14	14	14	0	100%	
Nontarget organisms	12	12	12^*a*^	0	100%	

^*a*^ *G. otitidis* was identified as *M. sediminis*, but this was not counted as an incorrect identification because the sample correctly failed acceptability criteria.

^*b*^ Evaluable sputa included total samples tested minus invalid samples.

^*c*^ N/A, Not applicable.

Deeplex Myc-TB also detects mutations associated with 13 anti-tuberculosis drugs (i.e., the resistotype), and its performance was compared to reference standards. For acquired isolates, published drug susceptibility results from reputable sources (e.g., BEI Resources, ATCC, and BCCM Culture Collections) were used where available. In addition, methods such as agar proportion, broth proportion, and real-time PCR were used for primary and/or discrepancy testing (Table S1).

For isolates, 31/532 (5.8%) susceptibility results were initially discordant between Deeplex and reference standards methods; after discrepant testing, 16/532 (3.0%) remained discordant (Table S4). For sputum samples, 28/520 (5.4%) results were initially discordant with reference standards, and after discrepant testing, 13/520 (2.5%) remained discordant (Table S5). Performance results for Deeplex Myc-TB resistotypes prior to discrepancy analysis are presented in Tables S4 and S5. Performance data for resistotypes after discrepancy testing showed that, overall, the categorical agreement of Deeplex Myc-TB resistotypes compared to reference methods was 91.9% (489/532 antibiotic results; 95% CI = 89.3%–94.0%) for isolates and 90.8% (472/520 antibiotic results; 95% CI = 88.0%–93.0%) for sputum samples (Tables 5 and 6). When the variants of unknown significance (VUS) were excluded, categorical agreement increased to 96.8% (489/505; 95% CI = 94.9%–98.1%) for isolates (Table 5) and 97.3% (472/485; 95% CI = 95.4%–98.5%) for sputa (Tables 5 and 6; see Tables S4 and S5 for data prior to discrepant analysis).

**TABLE 5:** Categorical agreement of the Deeplex Myc-TB resistotype on isolates compared to reference standard methods (results shown are after discrepant analysis; see Table S4 for results prior to discrepant analysis)^*a*^

Antibiotic	True S	True R	False S	False R	VUS	Total tested	Categorical agreement		Categorical agreement, after excluding VUS	
Rifampin	30	8	0	0	1	39	38/39	97.4%	38/38	100.0%
Isoniazid (Low CC)	20	12	3	1	3	39	32/39	82.1%	32/36	88.9%
Isoniazid (High CC)	23	12	0	1	3	39	35/39	89.7%	35/36	97.2%
Pyrazinamide	23	13	0	1	2	39	36/39	92.3%	36/37	97.3%
Streptomycin	26	9	0	0	4	39	35/39	89.7%	35/35	100.0%
Ethambutol	30	1	0	6	2	39	31/39	79.5%	31/37	83.8%
Fluoroquinolones	32	6	0	0	1	39	38/39	97.4%	38/38	100.0%
Capreomycin	34	3	0	1	1	39	37/39	94.9%	37/38	97.4%
Amikacin	34	4	0	0	1	39	38/39	97.4%	38/38	100.0%
Kanamycin	33	4	0	0	2	39	37/39	94.9%	37/37	100.0%
Ethionamide	31	3	1	0	4	39	34/39	87.2%	34/35	97.1%
Linezolid	35	2	0	0	1	38	37/38	97.4%	37/37	100.0%
Clofazimine	33	3	1	0	1	38	36/38	94.7%	36/37	97.3%
Bedaquiline	22	3	1	0	1	27	25/27	92.6%	25/26	96.2%
TOTAL	406	83	6	10	27	532	489/532	91.9%	489/505	96.8%

^*a*^ S, susceptible; R, resistant; VUS, variants of unknown significance; CC, critical concentration.

**TABLE 6:** Categorical agreement of the Deeplex Myc-TB resistotype on sputa compared to reference standard methods (results shown are after discrepant analysis; see Table S5 for results prior to discrepant analysis)^*a*^

Antibiotic	True S	True R	False S	False R	VUS	Total tested	Categorical agreement		Categorical agreement, after excluding VUS	
Rifampin	30	5	0	1	2	38	35/38	92.1%	35/36	97.2%
Isoniazid (Low CC)	18	12	3	0	5	38	30/38	78.9%	30/33	90.9%
Isoniazid (High CC)	21	12	0	0	5	38	33/38	86.8%	33/33	100.0%
Pyrazinamide	25	12	0	1	3	41	37/41	90.2%	37/38	97.4%
Streptomycin	25	9	0	0	3	37	34/37	91.9%	34/34	100.0%
Ethambutol	29	1	0	6	2	38	30/38	78.9%	30/36	83.3%
Fluoroquinolones	30	7	0	0	3	40	37/40	92.5%	37/37	100.0%
Capreomycin	32	3	0	1	0	36	35/36	97.2%	35/36	97.2%
Amikacin	32	4	0	0	1	37	36/37	97.3%	36/36	100.0%
Kanamycin	31	4	0	0	2	37	35/37	94.6%	35/35	100.0%
Ethionamide	29	3	0	0	5	37	32/37	86.5%	32/32	100.0%
Linezolid	37	2	0	0	1	40	39/40	97.5%	39/39	100.0%
Clofazimine	32	3	1	0	2	38	35/38	92.1%	35/36	97.2%
Bedaquiline	21	3	0	0	1	25	24/25	96.0%	24/24	100.0%
TOTAL	392	80	4	9	35	520	472/520	90.8%	472/485	97.3%

^*a*^ S, susceptible; R, resistant; VUS, variants of unknown significance; CC, critical concentration.

**TABLE 7:** Correlation of variants of unknown significance (VUS) with phenotypic susceptibility results

	Isolates		Sputum	
Antibiotic	Total VUS	Number (proportion) of VUS that were phenotypically susceptible	Total VUS	Number (proportion) of VUS that were phenotypically susceptible
Rifampin	1	1 (100%)	2	2 (100%)
Isoniazid (Low CC)	3	3 (100%)	5	5 (100%)
Isoniazid (High CC)	3	3 (100%)	5	5 (100%)
Pyrazinamide	2	2 (100%)	3	3 (100%)
Streptomycin	4	3 (75%)	3	2 (67%)
Ethambutol	2	2 (100%)	2	2 (100%)
Fluoroquinolones	1	1 (100%)	3	3 (100%)
Capreomycin	1	1 (100%)	0	0 (N/A)
Amikacin	1	1 (100%)	1	1 (100%)
Kanamycin	2	2 (100%)	2	2 (100%)
Ethionamide	4	4 (100%)	5	4 (80%)
Linezolid	1	0 (0%)	1	0 (0%)
Clofazimine	1^*a*^	0 (0%)	2	2 (100%)
Bedaquiline	1^*a*^	0 (0%)	1	1 (100%)
TOTAL	27	23 (85.2%)	35	32 (91.4%)

^*a*^ Laboratory-derived isolate that was phenotypically resistant to both CFZ and BDQ; WGS revealed a G103A mutation, which published reports indicate can be associated with resistance (13, 14).

Most VUS results were associated with phenotypic susceptibility; 85.2% (23/27; 95% CI = 66.9%–94.7%) of isolates and 91.4% of sputa (32/35; 95% CI = 76.9%–97.8%) with VUS results for an antibiotic were phenotypically susceptible for that antibiotic (Table 7). However, there were exceptions. For instance, one lab-derived isolate that was BDQ-resistant and CFZ-susceptible was classified as a BDQ-susceptible by Deeplex. Discrepancy testing was performed using WGS, which found that the strain carried a mutation in Rv1305 (*atpE*) that resulted in an A63P substitution, which is known to confer high-level resistance to BDQ (10–12, 15). A second strain that was phenotypically resistant to both BDQ and CFZ was reported as VUS for both antibiotics by Deeplex, with a G103A substitution identified in Rv0678 (*mmpR5*). Discrepancy testing using WGS detected the same mutation. Though this mutation is not currently classified as being associated with resistance in the World Health Organization (WHO) catalog (16), published reports indicate that it is associated with resistance (13, 14).

Per the user manual, the LOD is 100–1,000 mycobacterial genomes/PCR from extracted DNA. An initial range-finding assay around 100–1,000 CFU/PCR was performed for isolates and sputum samples, with a representative *M. tuberculosis* and an NTM clinical strain. Deeplex Myc-TB correctly identified MTBC at lower concentrations—30 CFU/PCR for pure isolates and 25 CFU/PCR from sputum—but resistotype accuracy was less reliable at lower concentrations (Tables S6 to S8). Therefore, range-finding results suggested that the best performance for both identification and resistotype for MTBC was 61–243 CFU/PCR for isolates and ~78–114 CFU/PCR for sputum samples (Tables S6 and S7).

Range-finding was followed by LOD testing, where testing was confirmed at a single concentration using 20 replicates in order to achieve ≥95% detection for both identification and resistotype. Both *M. tuberculosis* and an NTM were tested using two operators. For isolates, the LOD was confirmed to be at least 243 CFU/PCR; the most accurate LOD for isolates is likely less than 243 CFU (i.e., between 61–243 CFU based on manufacturer and our range-finding data), but further testing at a lower concentration was unable to be tested within the scope of the project. LOD for sputum samples was performed using 114 CFU/PCR, as determined by range-finding testing, to capture the ideal concentration for identification and resistotype (Table S8). For *M. abscessus* subsp. *bolletii*, the LOD was 110 CFU/PCR from isolates and 100 CFU/PCR from sputum (Table S8) although detection may occur below that level based on range-finding results (Tables S6 and S7).

## DISCUSSION

Molecular genetic assays for identifying mycobacterial species and predicting drug susceptibility in MTBC can improve diagnostic reliability while reducing costs and turnaround time (13–17). WGS, although comprehensive, is challenging to implement routinely because of biomass requirements, the need for pure isolates rather than primary specimens, and the substantial bioinformatic expertise required (18). Many of these limitations may be overcome by using commercial tNGS/WGS with bioinformatics pipelines for organism identification and detection of resistance mutations, especially those that can be used on both isolates and directly from primary specimens.

In this study, Deeplex Myc-TB demonstrated 100% specificity for mycobacterial targets from both isolates and sputum. Among isolates, sensitivity for species identification was 90%–100% for most target organisms, and reproducibility was high for both intra-assay (100%, 287/287 data points) and inter-assay (100%, 622/622) precision testing (data not shown). Two limitations were noted when testing isolates: poor discrimination between *M. bovis* and *M. bovis* BCG, and the inability to identify rare SGM, such as *M. conspicuum* and *M. parascrofulaceum*. Prior work with a larger sample size of *M. abscessus* isolates (*n* = 64) showed accurate subspecies differentiation (*bolletii, abscessus*, and *massiliense*) (19). Our study confirmed these findings for isolates, but detection and identification of *M. abscessus* directly from sputum was less robust.

In sputum, Deeplex Myc-TB performed markedly better for MTBC than for NTM, as more than half of the culture-positive sputum samples containing NTM were invalid. Among the few remaining evaluable samples (only 3 RGM and 10 SGM), the sensitivity for NTM identification was low (67%–70%). In contrast, Deeplex Myc-TB detected 97.6% (42/43) of culture-positive MTBC sputum samples, although, as with the isolate results, the *M. bovis* vs *M. bovis* BCG distinction remained problematic. As with isolate testing, reproducibility from sputum was high for both intra-assay (100%, 273/273) and inter-assay (100%, 570/570) precision testing (data not shown). These findings suggest that the assay is optimized for direct MTBC detection from sputum but is less reliable for direct NTM detection.

The Deeplex Myc-TB resistotype showed a high level of agreement with reference phenotypic susceptibility testing, consistent with previous reports (5, 20, 21). The assay accurately detected common MTBC resistance-associated mutations across 13 first- and second-line anti-tuberculosis drugs, providing considerable benefits for diagnostic speed and accuracy compared with phenotypic testing. In recognition of these advantages, the WHO conditionally recommends tNGS assays—including Deeplex Myc-TB, Ampore-TB (Oxford Nanopore Diagnostics), and TB-Seq (ShengTing Medical Technology)—for comprehensive MTBC drug-resistance detection (3). Early identification of antibiotic resistance allows clinicians to tailor therapies sooner, potentially shortening treatment duration and improving patient outcomes, although the cost of tNGS remains a consideration (22).

Categorical agreement between Deeplex resistotypes and phenotypic antimicrobial susceptibility testing (pAST) exceeded 90% for isolates and 95% for sputum after discrepant analysis and exclusion of VUS, the latter of which cannot be phenotypically adjudicated. Two drugs demonstrated lower agreement: isoniazid at the lower critical concentration (88.9% in isolates; 90.9% in sputum) and ethambutol (83.8% in isolates; 83.3% in sputum). However, WHO guidance acknowledges that low-level isoniazid resistance may be overcome with higher-dose therapy and that molecular results may better correlate with clinical outcomes (9). Since the recommendations suggest that molecular results may supersede phenotypic isoniazid testing at the lower level, the poor correlation between the tests is of less concern. Likewise, phenotypic testing for ethambutol is notoriously variable and is not recommended by WHO due to inconsistent performance (9, 11). Therefore, Deeplex Myc-TB therefore may serve as a preferable alternative to phenotypic testing for these two drugs.

Most VUS detected by Deeplex Myc-TB were associated with phenotypic susceptibility, though not always. For example, Deeplex Myc-TB detection of BDQ-resistance is limited in that it only targets Rv0678 (*mmpR5*), the most commonly mutated gene in BDQ-resistant clinical isolates. On the other hand, it does not detect less frequent but high-level BDQ-resistance mutations in Rv1305 (*atpE*), or other resistance-associated mutations in Rv2535c (*pepQ*) and Rv0676c/Rv0677c (*mmpL5/mmpS5*) (5, 23). Although these mutations remain relatively rare, the findings highlight the importance of complementing Deeplex Myc-TB with either phenotypic testing or WGS for antibiotics like BDQ and CFZ, and they underscore the potential value of expanding tNGS gene targets for more comprehensive predictions. Laboratories should, therefore, advise users that additional molecular or phenotypic data may be required (24).

This study has several limitations. For instance, many MTBC and NTM sputum specimens were contrived by spiking into artificial sputum matrix because clinical sputum volumes were limited and our laboratory receives few primary MTBC or rare NTM specimens. Artificial sputum has been used successfully in prior studies (25, 26), but it may not fully replicate the complexity of clinical sputum which may contain factors (e.g., PCR inhibitors) that could interfere with DNA amplification. As a result, it is possible that MTBC identification was more successful than NTM identification in this study because most MTBC samples were spiked in artificial sputum matrix whereas most NTM samples were clinical samples. Another explanation for the low proportion of evaluable NTM specimens may be related to their low bacillary load (i.e., AFB smear negative or 1+). Evaluation of additional primary specimen types, such as BAL fluid and tissue samples, as well as performance from MGIT broth vs solid media, could potentially offer better NTM identification.

Importantly, our extraction method deviated from the protocol outlined in the manufacturer’s instructions, as we employed a DNA extraction procedure that would help streamline our turnaround times. Therefore, incorporating a bead beating step may have affected our assay performance although previous molecular testing in our laboratory suggests that the extraction steps utilized for this study are efficient for mycobacteria.

Another limitation of our study is that we were unable to test large numbers of isolates resistant to ethambutol, capreomycin, ethionamide, linezolid, clofazimine, and bedaquiline due to their limited availability. Finally, a full analysis of implementation was not performed, but the cost and technical expertise required for this test is a significant consideration and can be affected by automated vs manual extraction methods, reagents used, and technician time. On the other hand, the extraction, library preparation, and sequencing components of the Deeplex Myc-TB assay can be performed for up to 48 samples in just 2–3 days, compared to phenotypic methods which can take 1–3 months to perform.

Overall, Deeplex Myc-TB demonstrated strong performance for mycobacterial identification from isolates, high accuracy for MTBC identification and resistotyping directly from sputum, and excellent agreement between resistotypes and reference susceptibility data. Although differentiation of *M. bovis* from BCG was imperfect and NTM detection from sputum was low, performance for MTBC was robust. Therefore, the Deeplex Myc-TB tNGS assay represents a valuable tool for clinical laboratories, particularly for rapid MTBC drug-susceptibility prediction from primary specimens. Its implementation could meaningfully improve turnaround times and provide earlier, more accurate therapeutic guidance for patients with tuberculosis.

## SUPPLEMENTAL MATERIAL

Supplemental tables jcm.00106-26-s0001.docx Tables S1 to S9.

ASM does not own the copyrights to Supplemental Material that may be linked to, or accessed through, an article. The authors have granted ASM a non-exclusive, world-wide license to publish the Supplemental Material files. Please contact the corresponding author directly for reuse.
