# Supplementary material for: Performance of a targeted next generation sequencing assay for mycobacterial identification and drug resistance from sputum and isolates
Source: J Clin Microbiol. 2026 Jun 12;64(7):e00106-26. doi: 10.1128/jcm.00106-26 (PMC13343929; doi:10.1128/jcm.00106-26)
Supplement: Supplemental tables — Tables S1 to S9. [file jcm.00106-26-s0001.docx]

# **SUPPLEMENTARY TABLES**

Table S1. Description of Phenotypic Antibiotic Susceptibility Testing Methods Used

| **Antibiotic** | **Primary phenotypic test method (number of isolates tested)** | **Test method used for discrepancy analysis (number of isolates tested)** | **Concentrations tested (µg/ml) on agar** | **Concentrations tested (µg/ml) on broth** |
| --- | --- | --- | --- | --- |
| Rifampin | Agar proportion (39) | MTB/Rif (GeneXpert, Cepheid), broth proportion (3) | 1^a^ | 0.5, 1, 2 |
| Isoniazid (Low) | Agar proportion (39) | Broth proportion (6) | 0.2 | 0.025, 0.05, 0.1, 0.2, 0.4, 0.8 |
| Isoniazid (High) | Agar proportion (39) | Broth proportion (3) | 1 |  |
| Pyrazinamide | Broth proportion (39) | Repeat testing (2) | - | 50, 100, 200, 400 |
| Streptomycin | Agar proportion (39) | Broth proportion (1) | 2, 4 | 1, 2, 4, 8 |
| Ethambutol | Agar proportion (39) | Broth proportion (6) | 7.5 | 2.5, 5, 10 |
| Fluoroquinolones (levofloxacin) | Broth proportion (39) | Repeat testing (1) | - | 0.5, 1, 2 |
| Capreomycin | Agar proportion (39) | Broth proportion (3) | 10 | 2, 4, 8 |
| Amikacin | Agar proportion (39) | Repeat testing (0) | 6 |  |
| Kanamycin | Agar proportion (39) | Repeat testing (0) | 6 |  |
| Ethionamide | Agar proportion (39) | Broth proportion (2) | 10 | 1, 2, 4, 8 |
| Linezolid | Broth proportion (38) | Broth proportion (0) | - | 0.5, 1, 2, 4, 8 |
| Clofazimine | Agar dilution (38) | Repeat testing (3) | 0.12, 0.125, 0.5 | - |
| Bedaquiline | Broth microdilution (22) and BCCM results (5) | Agar proportion (1) | 0.25 | 0.015, 0.03, 0.06, 0.5, 1 |

^a^ Tested on 7H11; BCCM = Belgian Coordinated Collections of Microorganisms

Table S2. Number of invalid isolate results by failure type

| **Organism type** | **Total tested** | **Number invalid due to low Q30 score (<80%)** | **Number invalid due to poor extraction (Qubit values <0.2)** | **Number invalid due to insufficient mycobacterial targets detected (Quality Score = ND)** | **Number invalid due to low Average Coverage Depth (<40x)** | **Number invalid due to failure of any criteria** | **Invalid rate before repeat testing** |
| --- | --- | --- | --- | --- | --- | --- | --- |
| MTBC | 42 | 0 | 0 | 1^ | 0 | 1 | 2.4% |
| NTM | 21 | 0 | 0 | 1* | 0 | 1 | 4.8% |
| Negatives/ nontarget organisms | 15 | 0 | 3 | 15 | 15 | 15 | 100% |
| **Total** | **78** | **0** | **3** | **17** | **15** | **17** | **N/A** |

^ Testing was repeated but still resulted in a poor Qubit and quality score (ND), likely due to poor growth of the isolate.

* Repeat testing was performed and resulted in a sufficient quality score upon repeat.

Table S3. Number of invalid sputum results by failure type

| **Organism type** | **Total tested** | **Number invalid due to low Q30 score (<80%)** | **Number invalid due to poor extraction (Qubit values <0.2)** | **Number invalid due to insufficient mycobacterial targets detected (Quality Score = ND)** | **Number invalid due to low Average Coverage Depth (<40x)** | **Number invalid due to failure of any criteria** | **Invalid rate before repeat testing** |
| --- | --- | --- | --- | --- | --- | --- | --- |
| MTBC | 43 | 0 | 0 | 1 | 1 | 1 | 2.3% |
| NTM | 32 | 0 | 3 | 16 | 7 | 19 | 59.4% |
| Mixed | 1 | 0 | 0 | 0 | 0 | 0 | 0.0% |
| Negatives/ nontarget specimens | 26 | 0 | 24 | 25 | 21 | 26 | 100.0% |
| **Total** | **102** | **0** | **27** | **42** | **29** | **46** | **N/A** |

Table S4. Categorical Agreement of the Deeplex Resistotype on Isolates Compared to Reference Standard Methods (Before Discrepant Analysis)

| **Antibiotic** | **True S** | **True R** | **False**  **S** | **False R** | **VUS** | **Total** | **Comments** |
| --- | --- | --- | --- | --- | --- | --- | --- |
| Rifampin | 30 | 5 | 0 | 3^a^ | 1 | **39** | ^a^ 3 out of 3 originally sensitive by AP but confirmed rifampin resistance mutation(s) by MTB/Rif (GeneXpert, Cepheid). |
| Isoniazid (Low CC) | 20 | 10 | 3^b^ | 3^c^ | 3 | **39** | ^b^ 3 out of 3 were originally sensitive by AP but repeated as resistant. ^c^ 2 out of 3 were originally sensitive by AP but resistant by discrepant testing using BM |
| Isoniazid (High CC) | 23 | 10 | 0 | 3^d^ | 3 | **39** | ^d^ 2 out of 3 were originally sensitive by AP but resistant by discrepant testing using BM |
| Pyrazinamide | 22 | 13 | 1^e^ | 1^f^ | 2 | **39** | ^e^ Originally resistant by BM, but sensitive upon repeat. ^f^ repeated as sensitive twice by BM |
| Streptomycin | 26 | 8 | 0 | 1^g^ | 4 | **39** | ^g^ Originally sensitive by AP, but resistant upon repeat testing with BM |
| Ethambutol | 30 | 1 | 0 | 6^h^ | 2 | **39** | ^h^ All 6 were sensitive by AP and discrepant testing with BM |
| Fluoroquinolones | 31 | 6 | 1^i^ | 0 | 1 | **39** | ^i^ Originally resistant by BM, but repeated as sensitive with BM |
| Capreomycin | 34 | 1 | 0 | 3^j^ | 1 | **39** | ^j^ Originally sensitive by AP; upon repeat 2 tested as resistant and 1 was sensitive |
| Amikacin | 34 | 4 | 0 | 0 | 1 | **39** |  |
| Kanamycin | 33 | 4 | 0 | 0 | 2 | **39** |  |
| Ethionamide | 30 | 3 | 2^k^ | 0 | 4 | **39** | ^k^ 1 originally resistant by AP; repeated by BM as sensitive. |
| Linezolid | 35 | 2 | 0 | 0 | 1 | **38** |  |
| Clofazimine | 32^l^ | 2 | 2^m^ | 1^n^ | 1^o^ | **38** | ^l^ 2 out of 32 sensitive isolates were lab-derived; ^m^ 1 originally resistant by AD; repeated by BM as sensitive, while the other one remained resistant by AD ^n^ 1 originally sensitive by AD; repeated by BM as resistant. ^o^ 1 VUS isolate was lab-derived; WGS confirmed resistance conferred by a *rv0678* mutation (G103A) |
| Bedaquiline | 22^p,^ | 3 | 1^q^ | 0 | 1^r^ | **27** | ^p^ 1 out of 22 sensitive isolates was lab-derived; WGS confirmed sensitivity to BDQ. ^q^ 1 lab-derived isolate was resistant by AP; WGS confirmed an *atpE* (A63P) mutation for BDQ resistance. ^r^ 1 VUS isolate was lab-derived; WGS confirmed resistance conferred by a *rv0678* mutation (G103A) |
| **Total** | **402** | **72** | **10** | **21** | **27** | **532** | All false S and false R results underwent discrepancy testing (see Table 5 for final results) |

S = susceptible; R = resistant; VUS = variants of unknown significance; CC = critical concentration; WGS = whole genome sequencing; BM = broth microdilution; AP = agar proportion

Table S5. Categorical agreement of the Deeplex resistotype on sputum compared to reference standard testing on isolates grown from culture (Before discrepant analysis)

| **Antibiotic** | **True S** | **True R** | **False**  **S** | **False R** | **VUS** | **Total** | **Comments** |
| --- | --- | --- | --- | --- | --- | --- | --- |
| Rifampin | 27 | 5 | 0 | 4^a^ | 2 | **38** | ^a^ 3 out of 4 originally sensitive by phenotypic methods but confirmed rifampin resistance mutation(s) by MTB/Rif (GeneXpert, Cepheid). |
| Isoniazid (Low CC) | 18 | 10 | 3 | 2^b^ | 5 | **38** | ^b^ 2 out of 2 were originally sensitive by AP but resistant by discrepant testing using BM |
| Isoniazid (High CC) | 21 | 10 | 0 | 2^c^ | 5 | **38** | ^c^ 2 out of 2 were originally sensitive by AP but resistant by discrepant testing using BM |
| Pyrazinamide | 24 | 12 | 1^d^ | 1^e^ | 3 | **41** | ^d^ Originally resistant by BM, but sensitive upon repeat;  ^e^ Repeated as sensitive twice by BM |
| Streptomycin | 25 | 8 | 0 | 1^f^ | 3 | **37** | ^f^ Originally sensitive by AP, but resistant upon repeat testing with BM |
| Ethambutol | 29 | 1 | 0 | 6^g^ | 2 | **38** | ^g^ All 6 were sensitive by AP and discrepant testing with BM |
| Fluoroquinolones (levofloxacin) | 29 | 7 | 1^h^ | 0 | 3 | **40** | ^h^ Originally resistant by BM, but repeated as sensitive with BM |
| Capreomycin | 32 | 1 | 0 | 3^i^ | 0 | **36** | ^i^ Originally sensitive by AP; Upon repeat 2 tested as resistant and 1 was sensitive |
| Amikacin | 32 | 4 | 0 | 0 | 1 | **37** |  |
| Kanamycin | 31 | 4 | 0 | 0 | 2 | **37** |  |
| Ethionamide | 28 | 3 | 1^j^ | 0 | 5 | **37** | ^j^ 1 originally resistant by AP; repeated by BM as sensitive. |
| Linezolid | 37 | 2 | 0 | 0 | 1 | **40** |  |
| Clofazimine | 31 | 2 | 2^k^ | 1^l^ | 2 | **38** | ^k^ 1 originally resistant by AP; repeated by BM as sensitive. ^l^1 originally sensitive by AP; repeated by BM as resistant. |
| Bedaquiline | 21 | 3 | 0 | 0 | 1 | **25** |  |
| **Total** | **385** | **72** | **8** | **20** | **35** | **520** | All false S and false R results underwent discrepancy testing (see Table 6 for final results) |

S = susceptible; R = resistant; VUS = variant of unknown significance; CC = critical concentration; BM = broth microdilution; AP = agar proportion; WGS = whole genome sequencing

Table S6. Range-finding using Mycobacterium tuberculosis to estimate the limit of detection (LOD) in isolates, per acceptability criteria described in methods.

| **Organism type** | **CFU/ PCR reac-tion tested** | **Rep-**  **licate** | **Deeplex Identification** | **Resistotype*** | | | | | | | | | | | | |
| --- | --- | --- | --- | --- | --- | --- | --- | --- | --- | --- | --- | --- | --- | --- | --- | --- |
|  |  |  |  | **RIF** | **INH** | **PZA** | **EMB** | **SM** | **FQ** | **KAN** | **AMI** | **CAP** | **ETH** | **LIN** | **BDQ** | **CFZ** |
| MTBC | 4 | 1 | N/A |  |  |  |  |  |  |  |  |  |  |  |  |  |
|  |  | 2 | N/A |  |  |  |  |  |  |  |  |  |  |  |  |  |
|  | 8 | 1 | N/A |  |  |  |  |  |  |  |  |  |  |  |  |  |
|  |  | 2 | N/A |  |  |  |  |  |  |  |  |  |  |  |  |  |
|  | 15 | 1 | N/A |  |  |  |  |  |  |  |  |  |  |  |  |  |
|  |  | 2 | N/A |  |  |  |  |  |  |  |  |  |  |  |  |  |
|  | 30 | 1 | *Mycobacterium tuberculosis* complex | 1 | 1 | 1 | 1 | 1 | 1 | 1 | 0 | 0 | 1 | 1 | 0 | 0 |
|  |  | 2 | *Mycobacterium tuberculosis* complex | 1 | 1 | 1 | 1 | 1 | 1 | 0 | 0 | 1 | 1 | 0 | 1 | 1 |
|  | 61 | 1 | *Mycobacterium tuberculosis* complex | 1 | 1 | 1 | 1 | 1 | 1 | 1 | 0 | 0 | 1 | 0 | 0 | 0 |
|  |  | 2 | *Mycobacterium tuberculosis* complex | 1 | 1 | 1 | 1 | 1 | 1 | 0 | 0 | 0 | 1 | 0 | 0 | 0 |
|  | 243 | 1 | *Mycobacterium tuberculosis* complex | 1 | 1 | 1 | 1 | 1 | 1 | 0 | 0 | 0 | 1 | 0 | 0 | 0 |
|  |  | 2 | *Mycobacterium tuberculosis* complex | 1 | 1 | 1 | 1 | 1 | 1 | 0 | 0 | 0 | 1 | 0 | 0 | 0 |
|  | 608 | 1 | *Mycobacterium tuberculosis* complex | 1 | 1 | 1 | 1 | 1 | 1 | 0 | 0 | 0 | 1 | 0 | 0 | 0 |
|  |  | 2 | *Mycobacterium tuberculosis* complex | 1 | 1 | 1 | 1 | 1 | 1 | 0 | 0 | 0 | 1 | 0 | 0 | 0 |
|  | 1,217 | 1 | *Mycobacterium tuberculosis* complex | 1 | 1 | 1 | 1 | 1 | 1 | 0 | 0 | 0 | 1 | 0 | 0 | 0 |
|  |  | 2 | *Mycobacterium tuberculosis* complex | 1 | 1 | 1 | 1 | 1 | 1 | 0 | 0 | 0 | 1 | 0 | 0 | 0 |
|  | 3,650 | 1 | *Mycobacterium tuberculosis* complex | 1 | 1 | 1 | 1 | 1 | 1 | 0 | 0 | 0 | 1 | 0 | 0 | 0 |
|  |  | 2 | *Mycobacterium tuberculosis* complex | 1 | 1 | 1 | 1 | 1 | 1 | 0 | 0 | 0 | 1 | 0 | 0 | 0 |
|  | 10,950 | 1 | *Mycobacterium tuberculosis* complex | 1 | 1 | 1 | 1 | 1 | 1 | 0 | 0 | 0 | 1 | 0 | 0 | 0 |
|  |  | 2 | *Mycobacterium tuberculosis* complex | 1 | 1 | 1 | 1 | 1 | 1 | 0 | 0 | 0 | 1 | 0 | 0 | 0 |
| NTM | 55 | 1 | *Mycobacterium abscessus* subsp. *bolletii* |  |  |  |  |  |  |  |  |  |  |  |  |  |
|  |  | 2 | *Mycobacterium abscessus* subsp. *bolletii* |  |  |  |  |  |  |  |  |  |  |  |  |  |
|  | 110 | 1 | *Mycobacterium abscessus* subsp. *bolletii* |  |  |  |  |  |  |  |  |  |  |  |  |  |
|  |  | 2 | *Mycobacterium abscessus* subsp. *bolletii* |  |  |  |  |  |  |  |  |  |  |  |  |  |
|  | 550 | 1 | *Mycobacterium abscessus* subsp. *bolletii* |  |  |  |  |  |  |  |  |  |  |  |  |  |
|  |  | 2 | *Mycobacterium abscessus* subsp. *bolletii* |  |  |  |  |  |  |  |  |  |  |  |  |  |
|  | 1,100 | 1 | *Mycobacterium abscessus* subsp. *bolletii* |  |  |  |  |  |  |  |  |  |  |  |  |  |
|  |  | 2 | *Mycobacterium abscessus* subsp. *bolletii* |  |  |  |  |  |  |  |  |  |  |  |  |  |
|  | 3,300 | 1 | *Mycobacterium abscessus* subsp. *bolletii* |  |  |  |  |  |  |  |  |  |  |  |  |  |
|  |  | 2 | *Mycobacterium abscessus* subsp. *bolletii* |  |  |  |  |  |  |  |  |  |  |  |  |  |

CFU: Colony forming units. *Gray: not reported; Red-green-blue: colors reported by the Deeplex; Detected = 1, Not detected = 0

Table S7. Linearity testing using Mycobacterium tuberculosis to estimate the limit of detection (LOD) in sputum, per acceptability criteria described in methods.

| **Organism type** | **CFU/ PCR reaction tested** | **Rep-**  **licate** | **Deeplex Identification** | **Resistotype** | | | | | | | | | | | | | |
| --- | --- | --- | --- | --- | --- | --- | --- | --- | --- | --- | --- | --- | --- | --- | --- | --- | --- |
|  |  |  |  | **RIF** | **INH** | **PZA** | **EMB** | **SM** | **FQ** | **KAN** | **AMI** | **CAP** | **ETH** | **LIN** | **BDQ** | **CFZ** |  |
| MTBC | 4 | 1 | N/A |  |  |  |  |  |  |  |  |  |  |  |  |  |  |
|  |  | 2 | N/A |  |  |  |  |  |  |  |  |  |  |  |  |  |  |
|  | 25 | 1 | *Mycobacterium tuberculosis* complex | 1 | 1 | 1 | 1 | 1 |  | 0 | 0 | 0 | 1 | 0 | 1 | 1 |  |
|  |  | 2 | *Mycobacterium tuberculosis* complex | 1 | 1 | 1 | 1 |  | 1 |  |  |  | 1 | 0 | 0 | 0 |  |
|  | 46 | 1 | *Mycobacterium tuberculosis* complex | 1 | 1 | 1 | 1 | 1 | 1 | 1 | 1 | 1 | 1 | 0 | 0 | 0 |  |
|  |  | 2 | *Mycobacterium tuberculosis* complex | 1 | 1 | 1 | 1 | 1 | 1 | 0 | 0 | 0 | 1 | 0 | 1 | 1 |  |
|  | 78 | 1 | *Mycobacterium tuberculosis* complex | 1 | 1 | 1 | 1 | 1 | 1 | 0 | 0 | 0 | 1 | 0 | 0 | 0 |  |
|  |  | 2 | *Mycobacterium tuberculosis* complex | 1 | 1 | 1 | 1 | 1 | 1 | 0 | 0 | 0 | 1 | 0 | 0 | 0 |  |
|  | 114 | 1 | *Mycobacterium tuberculosis* complex | 1 | 1 | 1 | 1 | 1 | 1 | 0 | 0 | 0 | 1 | 0 | 0 | 0 |  |
|  |  | 2 | *Mycobacterium tuberculosis* | 1 | 1 | 1 | 1 | 1 | 1 | 0 | 0 | 0 | 1 | 0 | 0 | 0 |  |
| NTM | 0.1 | 1 | N/A |  |  |  |  |  |  |  |  |  |  |  |  |  |  |
|  |  | 2 | N/A |  |  |  |  |  |  |  |  |  |  |  |  |  |  |
|  | 0.7 | 1 | N/A |  |  |  |  |  |  |  |  |  |  |  |  |  |  |
|  |  | 2 | N/A |  |  |  |  |  |  |  |  |  |  |  |  |  |  |
|  | 3 | 1 | N/A |  |  |  |  |  |  |  |  |  |  |  |  |  |  |
|  |  | 2 | N/A |  |  |  |  |  |  |  |  |  |  |  |  |  |  |
|  | 6 | 1 | *Mycobacterium abscessus* subsp. *bolletii* |  |  |  |  |  |  |  |  |  |  |  |  |  |  |
|  |  | 2 | *Mycobacterium abscessus* subsp. *bolletii* |  |  |  |  |  |  |  |  |  |  |  |  |  |  |
|  | 41 | 1 | *Mycobacterium abscessus* subsp. *bolletii* |  |  |  |  |  |  |  |  |  |  |  |  |  |  |
|  |  | 2 | *Mycobacterium abscessus* subsp. *bolletii* |  |  |  |  |  |  |  |  |  |  |  |  |  |  |

CFU: Colony forming units. *Gray: not reported; Red-green-blue: colors reported by the Deeplex; Detected = 1, Not detected = 0

Table S8. Limit of detection (LOD) per Deeplex PCR reaction for identification and resistotype.

|  | **Isolates** | | **Sputum** | |
| --- | --- | --- | --- | --- |
|  | **Identification** | **Resistotype** | **Identification** | **Resistotype** |
| **MTBC** | 243 CFU (100%, 20/20)* | 243 CFU (100%, 260/260) | 114 CFU (100%, 19/19)^ | 114 (100%, 19/19)^ |
| **NTM** | 110 CFU (100%, 20/20) |  | 100 CFU (100%, 21/21)* |  |

* LOD is likely to be lower, since the linearity revealed correct MTBC isolate identification as low as 30 CFU/reaction and NTM sputum identification as low as 6 CFU/reaction. ^ One isolate had insufficient extraction (<0.2ng/µL) and sequencing results were not included.

*Table S9. Description of Sputum Samples*

| **#** | **Spiked into ASM** | **Amount spiked in** **(where applicable)** | **Smear Result** | **Group** | **Expected Organism** | **Deeplex identification (Best match)** |
| --- | --- | --- | --- | --- | --- | --- |
| 1 | Y | 2X LOD | +2 | MTBC | *Mycobacterium tuberculosis* | *Mycobacterium tuberculosis* complex |
| 2 | Y | 2X LOD | +3 | MTBC | *Mycobacterium bovis* BCG | *Mycobacterium tuberculosis* complex |
| 3 | Y | 2X LOD | +3 | MTBC | *Mycobacterium tuberculosis* complex | *Mycobacterium tuberculosis* complex |
| 4 | Y | 2X LOD | +2 | MTBC | *Mycobacterium tuberculosis* complex | *Mycobacterium tuberculosis* complex |
| 5 | Y | 2X LOD | +2 | MTBC | *Mycobacterium bovis* BCG | *Mycobacterium tuberculosis* complex |
| 6 | Y | 2X LOD | +2 | MTBC | *Mycobacterium tuberculosis* | *Mycobacterium tuberculosis* complex |
| 7 | Y | 5X LOD | +4 | MTBC | *Mycobacterium tuberculosis* | *Mycobacterium tuberculosis* complex |
| 8 | Y | 5X LOD | +4 | MTBC | *Mycobacterium tuberculosis* | *Mycobacterium tuberculosis* complex |
| 9 | Y | 5X LOD | +2 | MTBC | *Mycobacterium tuberculosis* | *Mycobacterium tuberculosis* complex |
| 10 | Y | 5X LOD | +3 | MTBC | *Mycobacterium bovis* BCG | *Mycobacterium tuberculosis* complex |
| 11 | Y | 7X LOD | +1 | MTBC | *Mycobacterium tuberculosis* | *Mycobacterium tuberculosis* complex |
| 12 | Y | 7X LOD | +1 | MTBC | *Mycobacterium tuberculosis* complex | *Mycobacterium tuberculosis* complex |
| 13 | Y | 7X LOD | +1 | MTBC | *Mycobacterium tuberculosis* complex | *Mycobacterium tuberculosis* complex |
| 14 | Y | 7X LOD | +1 | MTBC | *Mycobacterium tuberculosis* complex | *Mycobacterium tuberculosis* complex |
| 15 | Y | 7X LOD | Negative | MTBC | *Mycobacterium tuberculosis* complex | *Mycobacterium tuberculosis* complex |
| 16 | Y | 7X LOD | Negative | MTBC | *Mycobacterium tuberculosis* complex | *Mycobacterium tuberculosis* complex |
| 17 | Y | 10X LOD | +1 | MTBC | *Mycobacterium tuberculosis* | *Mycobacterium tuberculosis* complex |
| 18 | Y | 10X LOD | Negative | MTBC | *Mycobacterium tuberculosis* complex | *Mycobacterium tuberculosis* complex |
| 19 | Y | 10X LOD | +1 | MTBC | *Mycobacterium tuberculosis* | *Mycobacterium tuberculosis* complex |
| 20 | Y | 10X LOD | +3 | MTBC | *Mycobacterium tuberculosis* | *Mycobacterium tuberculosis* complex |
| 21 | Y | 10X LOD | Negative | MTBC | *Mycobacterium bovis* BCG | *Mycobacterium tuberculosis* complex |
| 22 | Y | 10X LOD | Negative | MTBC | *Mycobacterium tuberculosis* complex | *Mycobacterium tuberculosis* complex |
| 23 | Y | 10X LOD | Negative | MTBC | *Mycobacterium tuberculosis* complex | *Mycobacterium tuberculosis* complex |
| 24 | Y | 10X LOD | Negative | MTBC | *Mycobacterium tuberculosis* complex | *Mycobacterium tuberculosis* complex |
| 25 | Y | 7X LOD | Negative | MTBC | *Mycobacterium tuberculosis* complex | *Mycobacterium tuberculosis* complex |
| 26 | Y | 2X LOD | Negative | MTBC | *Mycobacterium tuberculosis* complex | *Mycobacterium tuberculosis* complex |
| 27 | N | Clinical sample | +1 | MTBC | *Mycobacterium tuberculosis* | *Mycobacterium tuberculosis* complex |
| 28 | N | Clinical sample | Negative | MTBC | *Mycobacterium tuberculosis* | *Mycobacterium tuberculosis* complex |
| 29 | Y | 5X LOD | +4 | MTBC | *Mycobacterium tuberculosis* | *Mycobacterium tuberculosis* complex |
| 30 | Y | 5X LOD | +3 | MTBC | *Mycobacterium tuberculosis* | *Mycobacterium tuberculosis* complex |
| 31 | Y | 2X LOD | +1 | MTBC | *Mycobacterium tuberculosis* | *Mycobacterium tuberculosis* complex |
| 32 | Y | 2X LOD | +4 | MTBC | *Mycobacterium tuberculosis* | *Mycobacterium tuberculosis* complex |
| 33 | Y | 5X LOD | +4 | MTBC | *Mycobacterium tuberculosis* | *Mycobacterium tuberculosis* complex |
| 34 | Y | 5X LOD | +4 | MTBC | *Mycobacterium tuberculosis* | *Mycobacterium tuberculosis* complex |
| 35 | Y | 2X LOD | +4 | MTBC | *Mycobacterium tuberculosis* | *Mycobacterium tuberculosis* complex |
| 36 | Y | 2X LOD | +4 | MTBC | *Mycobacterium tuberculosis* | *Mycobacterium tuberculosis* complex |
| 37 | Y | 2X LOD | +3 | MTBC | *Mycobacterium tuberculosis* | *Mycobacterium tuberculosis* complex |
| 38 | Y | 2X LOD | +3 | MTBC | *Mycobacterium bovis* | *Mycobacterium tuberculosis* complex |
| 39 | Y | 2X LOD | +4 | MTBC | *Mycobacterium tuberculosis* complex | *Mycobacterium tuberculosis* complex |
| 40 | Y | 2X LOD | +3 | MTBC | *Mycobacterium tuberculosis* complex | *Mycobacterium tuberculosis* complex |
| 41 | Y | 5X LOD | Negative | MTBC | *Mycobacterium bovis* | *Mycobacterium tuberculosis* complex |
| 42 | Y | 2X LOD | Negative | MTBC | *Mycobacterium africanum* | *Mycobacterium tuberculosis* complex |
| 43 | Y | 2X LOD | Negative | MTBC | *Mycobacterium africanum* |  |
| 44 | N | Clinical sample | +3 | SGM | *Mycobacterium intracellulare* | *Mycobacterium intracellulare :* 88.1 */ Mycobacterium tuberculosis* complex *:* 11.9 |
| 45 | N | Clinical sample | +1 | SGM | *Mycobacterium chimaera* |  |
| 46 | N | Clinical sample | +2 | SGM | *Mycobacterium intracellulare* | *Mycobacterium intracellulare* |
| 47 | N | Clinical sample | +1 | RGM | *Mycobacterium abscessus* subsp. *massiliense* | *Mycobacterium massiliense* |
| 48 | N | Clinical sample | +1 | Mixed | *Mycobacterium chimaera, Gordonia sputi,* and *Mycobacterium abscessus* subsp*. abscessus* | *Mycobacterium avium* complex *(*subsp *avium, paratuberculosis, silvaticum) :* 76.3 */ Mycobacterium chimaera :* 23.0 |
| 49 | N | Clinical sample | +, Un-quantified | SGM | *Mycobacterium gordonae* |  |
| 50 | N | Clinical sample | +, Un-quantified | SGM | *Mycobacterium intracellulare* |  |
| 51 | N | Clinical sample | +1 | SGM | *Mycobacterium intracellulare* | *Mycobacterium intracellulare :* 90.4 / *Mycobacterium intracellulare :* 9.6 |
| 52 | N | Clinical sample | +1 | SGM | *Myocbacterium avium* | *Mycobacterium avium* complex *(*subsp *avium, paratuberculosis, silvaticum)* |
| 53 | N | Clinical sample | +1 | RGM | *Mycobacterium abscessus* subsp*. massiliense* | *Mycobacterium massiliense* |
| 54 | N | Clinical sample | +, Un-quantified | SGM | *Mycobacterium intracellulare* |  |
| 55 | N | Clinical sample | +2 | RGM | *Mycobacterium abscessus* subsp. *abscessus* |  |
| 56 | N | Clinical sample | +2 | RGM | *Mycobacterium abscessus* subsp. *abscessus* | *Mycobacterium abscessus subsp abscessus* |
| 57 | N | Clinical sample | +1 | RGM | *Mycobacterium abscessus* subsp. *abscessus* | *Mycobacterium intracellulare :* 79.5 */ Mycobacterium intracellulare :* 20.0 |
| 58 | N | Clinical sample | +1 | SGM | *Mycobacterium chimaera* |  |
| 59 | N | Clinical sample | Negative | RGM | *Mycobacterium abscessus* subsp. *abscessus* |  |
| 60 | N | Clinical sample | Negative | SGM | *Mycobacterium intracellulare* |  |
| 61 | N | Clinical sample | Negative | RGM | *Mycobacterium abscessus* subsp. *abscessus* |  |
| 62 | N | Clinical sample | Negative | SGM | *Myocbacterium avium* | *Mycobacterium chimaera* |
| 63 | N | Clinical sample | Negative | SGM | *Mycobacterium intracellulare* | *Mycobacterium intracellulare* |
| 64 | N | Clinical sample | Negative | SGM | *Mycobacterium intracellulare* |  |
| 65 | N | Clinical sample | Negative | RGM | *Mycobacterium abscessus* subsp. *abscessus* |  |
| 66 | N | Clinical sample | Negative | SGM | *Myocbacterium avium* |  |
| 67 | N | Clinical sample | Negative | SGM | *Mycobacterium intracellulare* | *Mycobacterium phocaicum :* 84.3 */ Mycobacterium intracellulare :* 15.5 |
| 68 | N | Clinical sample | Negative | RGM | *Mycobacterium abscessus* subsp. *massiliense* |  |
| 69 | N | Clinical sample | Negative | SGM | *Mycobacterium intracellulare* | *Mycobacterium intracellulare* |
| 70 | N | Clinical sample | Negative | SGM | *Mycobacterium intracellulare* | *Mycobacterium intracellulare :* 84.2 */ Mycobacterium intracellulare :* 15.8 |
| 71 | N | Clinical sample | Negative | RGM | *Mycobacterium abscessus* subsp. *abscessus* |  |
| 72 | Y | 5X LOD | +3 | SGM | *Mycobacterium simiae* | *Mycobacterium simiae* |
| 73 | Y | 2X LOD | Negative | RGM | *Mycobacterium senegalense* |  |
| 74 | Y | 10X LOD | +2 | SGM | *Mycobacterium parascrofulaceum* | *Mycobacterium seoulense* |
| 75 | Y | 7X LOD | +1 | SGM | *Mycobacterium intermedium* | *Mycobacterium intermedium* |
| 76 | Y | 5X LOD | Negative | SGM | *Mycobacterium marinum* |  |
| 77 | N/A | N/A | Negative | Negatives | None |  |
| 78 | N/A | N/A | Negative | Negatives | None |  |
| 79 | N/A | N/A | Negative | Negatives | None |  |
| 80 | N/A | N/A | Negative | Negatives | None |  |
| 81 | N/A | N/A | Negative | Negatives | None |  |
| 82 | N/A | N/A | Negative | Negatives | None |  |
| 83 | N/A | N/A | Negative | Negatives | None |  |
| 84 | N/A | N/A | Negative | Negatives | None |  |
| 85 | N/A | N/A | Negative | Negatives | None |  |
| 86 | N/A | N/A | Negative | Negatives | None |  |
| 87 | N | Clinical sample | Negative | Nontarget | *Nocardia* |  |
| 88 | N | Clinical sample | Negative | Nontarget | *Gordonia otitidis* | *Mycobacterium sediminis* |
| 89 | Y | 10X LOD | Negative | Nontarget | *Nocardia cyriacigeorgica* |  |
| 90 | Y | 10X LOD | Negative | Nontarget | *Tsukamurella pulmonis* |  |
| 91 | Y | 10X LOD | Negative | Nontarget | *Nocardia brasiliensis* |  |
| 92 | Y | 10X LOD | Negative | Nontarget | *Tsukamurella tyrosinosolvens* |  |
| 93 | Y | 10X LOD | Negative | Nontarget | *Nocardia nova* |  |
| 94 | Y | 10X LOD | Negative | Nontarget | *Nocardia farcinica* |  |
| 95 | Y | 10X LOD | Negative | Nontarget | *Nocardia otitidiscaviarum* |  |
| 96 | Y | 10X LOD | Negative | Nontarget | *Nocardia veterana* |  |
| 97 | Y | 10X LOD | Negative | Nontarget | *Staphylococcus aureus* |  |
| 98 | Y | 10X LOD | Negative | Nontarget | *Enterococcus faecalis* |  |
| 99 | N/A | N/A | Negative | Negatives | None |  |
| 100 | N/A | N/A | Negative | Negatives | None |  |
| 101 | N/A | N/A | Negative | Negatives | None |  |
| 102 | N/A | N/A | Negative | Negatives | None |  |

ASM = artificial sputum matrix; LOD = Limit of Detection; MTBC: *M. tuberculosis* complex; RGM = rapidly growing mycobacteria; SGM = slowly growing mycobacteria; N/A = not applicable
